# Listeria meningitis complicated by hydrocephalus in an immunocompetent child: case report and review of the literature

**DOI:** 10.1186/s13052-020-00873-w

**Published:** 2020-08-02

**Authors:** Giacomo Brisca, Alberto La Valle, Claudia Campanello, Mattia Pacetti, Mariasavina Severino, Giuseppe Losurdo, Antonella Palmieri, Isabella Buffoni, Salvatore Renna

**Affiliations:** 1 Pediatric Emergency Unit, IRCCS Istituto Giannina Gaslini, via Gerolamo Gaslini 5, 16147 Genoa, Italy; 2 Department of Neurosurgery, IRCCS Istituto Giannina Gaslini, via Gerolamo Gaslini 5, 16147 Genoa, Italy; 3 Neuroradiology Unit, IRCCS Istituto Giannina Gaslini, via Gerolamo Gaslini 5, 16147 Genoa, Italy; 4 Infectious Disease Unit, IRCCS Istituto Giannina Gaslini, via Gerolamo Gaslini 5, 16147 Genoa, Italy; 5 Neonatal and Pediatric Intensive Care Unit, IRCCS Istituto Giannina Gaslini, via Gerolamo Gaslini 5, 16147 Genoa, Italy

**Keywords:** *Listeria monocytogenes*, Meningitis, Hydrocephalus, Brain MRI, Case report

## Abstract

**Background:**

*Listeria monocytogenes* is a Gram-positive bacteria transmitted to human by animal stools, contaminated water and food.

In children, *Listeria monocytogenes* typically affects newborns and immunocompromised patients often leading to invasive syndromes including sepsis, brain abscesses, meningitis, meningoencephalitis and rhombencephalitis.

In healthy and immunocompetent children, Listeria meningitis is rare, but can progress rapidly and may be associated with severe complications (hydrocephalus, ventriculitis, cranial nerves palsy and cerebrospinal abscesses) and high mortality rate.

**Case presentation:**

We describe a very uncommon case of meningoencephalitis due to *Listeria monocytogenes* in a 11-month-old immunocompetent girl. Cerebrospinal fluid (CSF) culture was positive on the second day. Antibiotic therapy was promptly started but the disease was complicated by neurological deterioration and decompensated hydrocephalus. The child required a very demanding pediatric and neurosurgical management and was discharged after 40 days without major sequelae.

**Conclusion:**

Listeria is difficult to isolate and it is not susceptible to first-line treatment for bacterial meningitis with extended-spectrum cephalosporins. Early recognition is therefore crucial for a positive outcome. Pediatricians have to perform close clinical monitoring of these children and be aware of possible complications.

A review of all cases of Listeria meningitis complicated by hydrocephalus in healthy children has been performed, to provide an overview on clinical features, treatment options and outcome.

## Introduction

*Listeria monocytogenes* is a gram-positive, facultative intracellular bacteria that typically affects pregnant women, newborns, immunocompromised and older adults [1]. It is widespread in nature and isolated in soil, dust, water and sewage, spreading to humans through contaminated water and food [2].

Ready-to-eat foods such as smoked fish, ready-to-eat deli meats and soft cheeses are often the source of Listeria infections as their long shelf life is conducive to bacterial growth [3].

In immunocompromised patients, Listeria can be responsible for invasive syndromes including sepsis, brain abscesses, meningitis, meningoencephalitis and rhomboencephalitis [4].

There are rare reports of Listeria meningitis in previously healthy and immunocompetent children, which may be associated with severe complications (hydrocephalus, ventriculitis, cranial nerves palsy and cerebrospinal abscesses) and high mortality rate [4, 5].

We describe an unusual case of Listeria meningoencephalitis complicated by hydrocephalus in an immunocompetent infant. We also reviewed the literature for Listeria meningitis cases complicated by hydrocephalus in healthy children, in order to evaluate clinical features, treatment and outcome.

## Case report

We present the case of a previously healthy, 11-month-old girl, fully immunized, admitted to our emergency department with 3 days history of high-grade fever (maximum axillary temperature 40.1 °C), vomiting, diarrhea, sudden onset of lethargy, and uncontrolled movements of right leg.

The child was not attending the nursery and she was only taking paracetamol for fever before admission.

She presented in poor general conditions, pale, with flash capillary refill, bulging fontanel, miotic pupils and left convergent squint. During clinical examination in emergency room, she developed generalized tonic-clonic seizure and was treated with IV midazolam with resolution.

Initial laboratory investigations showed elevated white blood cells (WBC) counts of 23,510/μL (77% neutrophils) and high level of inflammatory markers: C-reactive protein (CRP) was 5.76 mg/dL (normal values < 0.46 mg/dL), and procalcitonin (PCT) was 3.45 ng/mL (normal values < 0.5 ng/mL). Electrolytes, renal and hepatic function and blood clotting tests were normal.

**Fig. 1:**
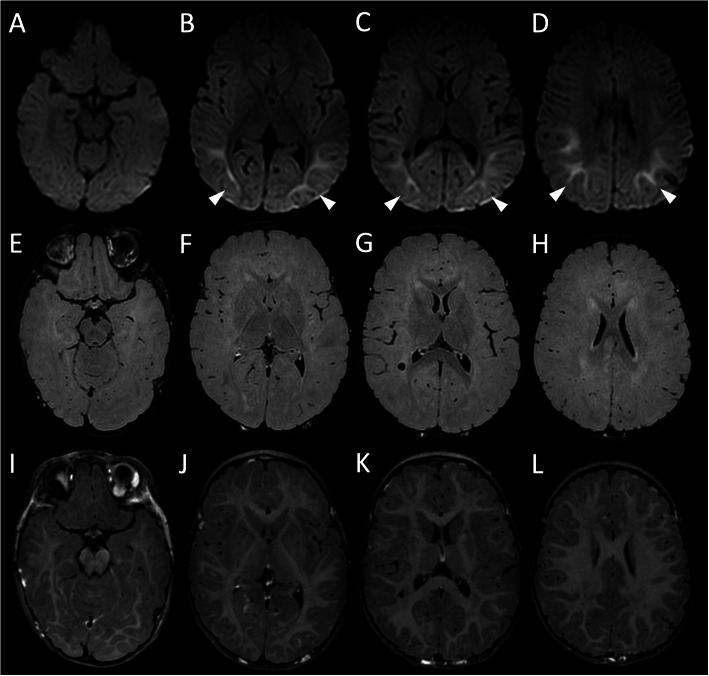
Brain MRI performed at clinical onset. **a**-**d** Axial diffusionweightedimages reveal restricted diffusion in the cortico-subcortical parieto-temporal regions (arrowhead), likely secondary to seizure-related changes. Axial contrast-enhanced FLAIR (**e**-**h**) and T1-weighted (**i-j**) images reveal normal ventricular size and absence of leptomeningeal contrast enhancement

An urgent brain magnetic resonance imaging (MRI) showed restricted diffusion with reduced ADC values at the level of the temporo-parietal regions, without leptomeningeal contrast-enhancement, consistent with seizure-related lesions (Fig. 1). Lumbar puncture was performed on the same day and cerebral-spinal fluid (CSF) was clear with neutrophilic pleocytosis (300 cells/μL with prevalence of polymorphonuclear cells), low glucose (8 mg/dl) and elevated protein levels (91 mg/dl). Real-time polymerase chain reaction (RT-PCR) for viruses (VZV, HSV 1–2, HHV 6, Adenovirus, EBV, CMV) and bacteria (*Streptococcus pneumoniae*, Mycoplasma *pneumoniae* and Neisseria *meningitidis*) resulted negative. Nasal and throat swab cultures, were negative. Stool cultures for Salmonella, Campylobacter, Rotavirus and Adenovirus and urine culture were all negative.

With clinical suspicion of meningoencephalitis, the little girl was empirically treated with intravenous ceftriaxone and acyclovir adding levetiracetam for seizures control. On day 2, no clinical improvement was observed as a persistent lethargy with poor response to stimulation, irritability, neck stiffness, lying position, right oral fissure asymmetry and global hypotonia was still present.

CSF staining showed gram positive bacilli. This finding, together with the low CSF glucose concentration, raised up the hypothesis of *Listeria monocytogenes* meningoencephalitis, confirmed, the following day, by CSF culture.

Ceftriaxone was switched to intravenous ampicillin (300 mg/kg/die in 3 times a die) and gentamicin (5 mg/kg/die). Acyclovir was stopped.

Immunological screening of cellular and humoral immunity, complement and blood iron level were normal. HIV test was negative.

**Fig. 2:**
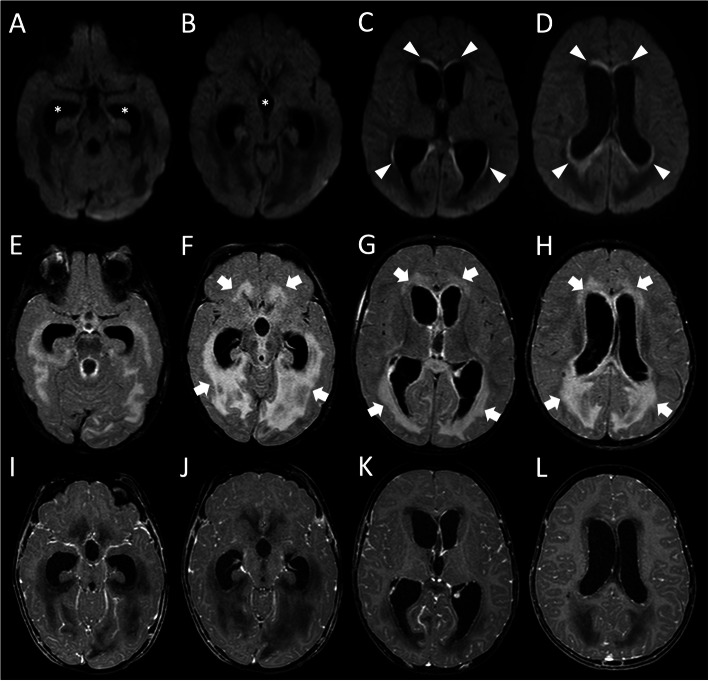
Brain MRI performed 6 days after clinical onset. **a**-**d** Axial diffusion weightedimages demonstrate resolution of signal alterations in the parieto-temporal regions, marked enlargement of the lateral and III ventricles (asterisks), and presence of linear restricted diffusion along the ependyma of the lateral ventricles (arrowheads), in keeping with ventricular ependymitis. Axial contrast-enhanced FLAIR (**e**-**h**) and T1-weighted (**i**-**j**) images show signal alterations at the level of the periventricular white matter (thick arrows), due to increased CSF transependymal reabsorption. Note the diffuse leptomeningeal contrast-enhancement at the level of both cerebral hemispheres associated with marked reduction of the cerebral subarachnoid spaces

After 4 days the child showed mild clinical improvement. She was afebrile with reduction of WBC count and normalization of inflammatory markers. However, on day 6, the neurological picture worsened again and a new cranio-spinal MRI revealed diffuse cerebrospinal leptomeningeal contrast-enhancement with signs of ventriculitis and severe decompensated communicating hydrocephalus (Fig. 2). The seizure-related brain changes were not present anymore.

She was urgently transferred to theatre for an external shunt placement in the right lateral ventricle (trans-frontal) and later admitted to the Neurosurgery Unit for the following management. CSF culture from ventricular draining was negative. Treatment with dexamethasone was started. Her neurological state improved in the following days, with regression of opisthotonus and irritability.

On day 27, a new head computed tomography (CT) scan showed persistence of ventriculomegaly with suspicion of trapped left ventricle that was not confirmed after intraventricular injection of contrast agent, showing no CSF obstruction at the level of left foramen of Monro. A new neurosurgical procedure was performed, introducing a second catheter in the frontal horn of the left lateral ventricle, connecting both catheters to a MIETHKE PaediGAV 4–24 shunt valve (B.Braun, Melsungen, AG) with subsequent peritoneal internalization.

**Fig. 3:**
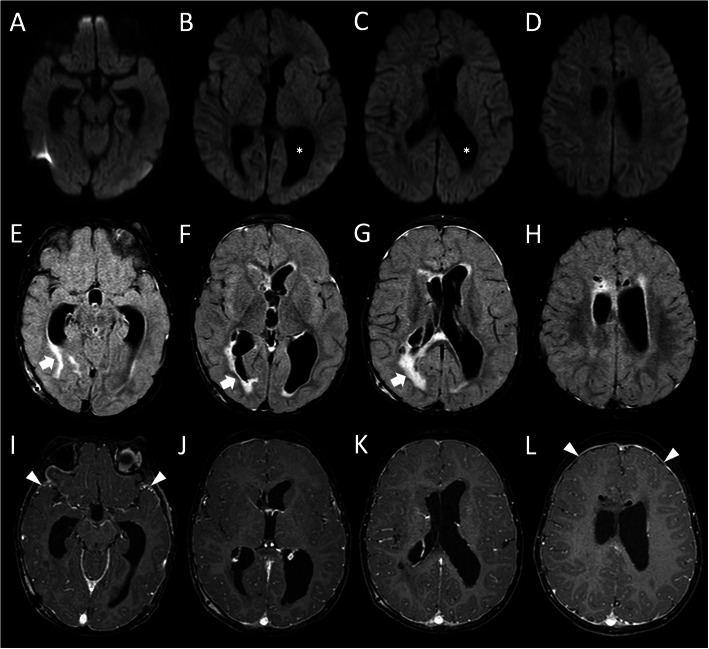
Brain MRI performed 1 month after clinical onset. **a**-**d** Axial diffusion weighted images show resolution of the signal abnormalities along the ventricular ependyma and reduction of the ventricular size after bilateral shunting procedures (asterisks). Axial contrast-enhanced FLAIR (**e**-**h**) and T1-weighted (**i**-**j**) images demonstrate marked reduction of the periventricular white matter signal alterations (thick arrows), and almost complete resolution of leptomeningeal contrast enhancement with re-expansion of the subarachnoid spaces. Note the presence of mild reactive dural contrast enhancement in the temporo-frontal regions (arrowheads)

Neurological conditions further improved, with resolution of left VI cranial nerve palsy and improvement of the contralateral palsy. Subsequent brain MRI showed a reduction of ventriculomegaly and almost complete resolution of leptomeningeal enhancement (Fig. 3).

The girl was discharged after 40 days of hospital treatment in good general condition with no major neurologic sequelae, except for a mild residual right lateral rectus palsy.

**Fig. 4:**
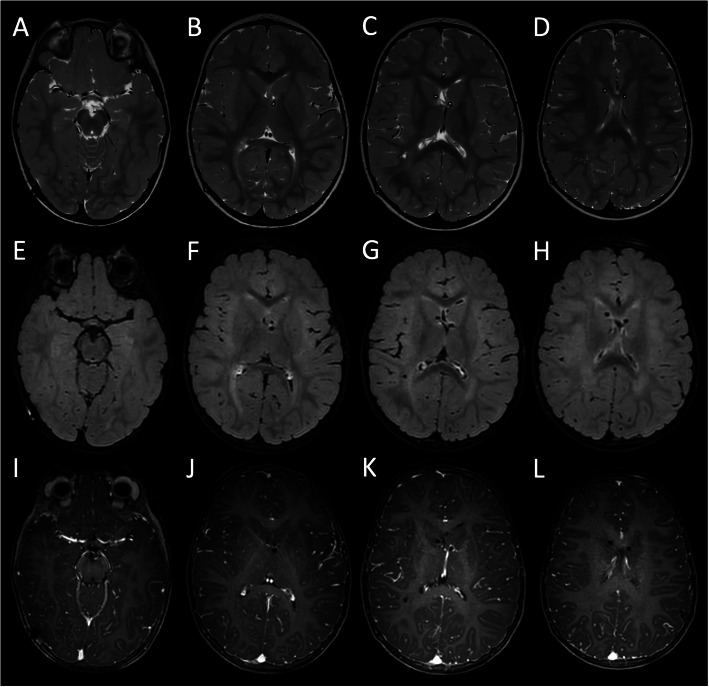
Brain MRI performed 6 months after clinical onset. Axial T2-weighted (**a**-**d**), contrast-enhanced FLAIR (**e**-**h**) and T1-weighted (**i**-**j**) images demonstrate complete resolution of the periventricular white matter signal alterations, leptomeningeal enhancement and reactive dural enhancement. Note the absence of brain lesions

On follow-up visit, 6 months after discharge, she appeared in good condition; the ventriculoperitoneal shunt was normally functioning. Brain MRI revealed complete resolution of the ventricular dilatation and leptomeningeal enhancement, and no brain lesions (Fig. 4).

Overall, the child was treated with Ampicillin for 26 days, Gentamicin for 15 days and then switched to Trimethoprim/sulfamethoxazole (TPM-SMX) for other 15 days.

The source of infection remains unclear: her parents denied consumption/ingestion of raw meat, cold cuts, precooked foods, unpasteurized milk or undercooked vegetables. Nevertheless, in the days before symptoms onset, we were told that the child was playing in a country setting eating tuft of grass contaminated by goat stools. Probably we may identify the gastrointestinal tract as the point of entry of bacteria.

## Discussion and conclusion

Meningitis is the most frequent presentation of *Listeria monocytogenes* infection [6]. Although rare in healthy children, beyond the neonatal period, its course can be rapid and aggressive with severe complications, such as acute hydrocephalus, and high mortality [4].

Our patient showed a very challenging clinical course, despite Listeria involvement was early identified and adequate antibiotic therapy was promptly introduced. Indeed, although the fever had disappeared and inflammatory markers were improving, the neurological deterioration lead to recognition of decompensated communicating hydrocephalus.

An increasing concern during bacterial meningitis is hydrocephalus. Commonly seen in subacute and chronic tuberculous or fungal meningitis, it can also occur in about 5% of adults with acute community-acquired meningitis and represents an independent unfavorable risk [7].

Listeria is reported as the second most common pathogen causing hydrocephalus, which develops in up to 14% of adult patients with Listeria meningitis [8]. Almost all patients have communicating hydrocephalus which reflects the failure of cerebrospinal resorption through arachnoid granulations due to severe infection [9].

A meta-analysis of the global and regional risks for disabling sequelae from bacterial meningitis found that since 2010 the overall increase in hydrocephalus in children older than 1 month with bacterial meningitis is 7.1% [10]. However, literature about hydrocephalus in Listeria meningitis in pediatric age is scarce and consists mostly of few case reports.

Reviewing literature, we identify nine previously healthy children with Listeria meningitis who developed hydrocephalus. Main clinical details are summarized in the Table 1. The median age was 3.3 years (range 7 months to 10 years), with six girls. The interval between the first symptoms and the diagnosis of hydrocephalus was around 8–10 days with a shortest recorded time of 5 days. The two children with the shortest interval (patient 8 and 9) were associated with the worst outcome (death in both cases). CSF findings were characterized by pleocytosis (range 150–6000 cells/mm^3^) with a prevalence of polymorphonuclear cells. Gram staining and culture confirmed their limited role, being negative in most cases (5/7 of cases with available data) at first lumbar puncture.

**Table 1:** Characteristics of reported children with hydrocephalus in Listeria meningitis.

***Patient and reference***	***Age (y/m), Gender*** ***(M/F)***	***CSF Gram Stain and culture positivization***	***CSF analysis at presentation***	***Therapy and duration***	***Time of hydrocephalus development***	***Neurosurgical management***	***Outcome***
1 present case	0 y, 11 m F	Gram stain positive Bacillus culture positive at day 2	WBC 300/mm^3^ Glu 8; Pr 91	Ampicillin 26 days, Gentamicin 15 days + TMP/SMX 15 days	- 10 days	*EVD and VPS*	Discharged after 40 days Persistence of right eye palsy
2 Xiaotang [16]	3 y, 3 m F	NA	WBC 162/mm^3,^ Glu and Pr unknown	Meropenem + Amikacina 3 days, Ampicillin + Meropenem 15 days	Unknown	*Unknown*	Discharge unknown Hearing lesion; development delay
3 Ben Shimol [17]	6 y, 0 m F	Gram stain unknown; culture positive on day 7	WBC 150/mm^3^ (N 65%)_;_ Glu 43; Pr 38	Ampicillin 28 days + gentamicin 12 days	- 9 days	EVD	Discharged after 34 days No sequelae
4 Montejo [18]	0 y,8 m F	Gram stain unknown; culture positive on day 3	WBC 240/mm^3^ (N 80%)_;_ Glu 35; Pr 57	Ampicillin + gentamicin 21 days	- 8 days	*EVD and VPS*	Discharged after unknown days Strabismus left eye.
5 Lee [19]	7 y, 0 m F	Gram stain negative on day 1; Gram stain positive Cocci on day 3	WBC 1500/mm^3^ (N 30%)_;_ Glu 25; Pr 117	Vancomycin + gentamicin+ ampicillin for unknown days	- 10 days	*EVD*	Discharged after 61 days No sequelae
6 Platnaris [20]	0 y, 7 m M	Gram stain negative on day 1; culture positive on day 3	WBC 2550/mm^3^ (N 80%)_;_ Glu 53; Pr 128,5	Gentamicin 14 days+ Ampicillin 21 days.	- 10 days	*EVD*	Discharged after 30 days No sequelae
7 Flodrops [21]	7 y, 0 m F	Gram stain negative on day 1; Culture positive on day 11	WBC 160/mm^3^ (N 5%)_;_ Glu 58; Pr 266	Vancomycin 8 days + amoxicillin 21 days	- 11 days	*EVD*	Discharged after unknown days No sequelae
8 Ulloa-Gutierrez [22]	3 y, 6 m M	Gram stain negative on day 1; Gram positive Cocci on day 3	WBC 755/mm^3^ (N 95%); Glu 21; Pr 266	Ampicillin + gentamicin 1 day until death	- 5 days	*EVD*	Death on day 4
9 Ulloa- Gutierrez [22]	6 y, 6 m M	Gram stain positive Coccobacilli on day 1; Culture positive on day 5	WBC 800/mm^3^ (N 60%); Glu 32; Pr 79	Ampicillin + gentamicin 3 days until death	- 7 days	*EVD*	Death on day 7
10 Ulloa- Gutierrez [22]	10 y, 0 m F	Negative Gram stain on day 1; Culture negative. Negative Gram stain on day 3; Culture positive on day 5	WBC 6000/mm^3^ (N 80%); Glu 62; Pr 121	Meropenem 22 days + Amikacin 10 days + Rifampin 14 days	- 8 days	*EVD*	Discharged after 28 days Upper left arm tremor and adiadochokynesis.

*Y* years, *M* months, *M* male, *F* female, *NA* not available, *Glu* glucose, *Pr* protein, *EVD* external ventricular drain, *VPS* ventricular-peritoneal shunt

Antibiotic treatment included ampicillin in combination with aminoglycoside in almost all patients. This is in accordance to ESCMID guidelines [11], although only a single in vitro study has shown this combination to be synergic [12]. Carbapenems have also been used in some cases (patient 2 and 10) but they were associated with higher mortality in long term therapy [13]. In our case, we used Ampicillin combined with Gentamicin, switching gentamicin to TPM/SMX after 15 days, to prevent toxicity aminoglycoside-related. TPM/SMX has a good penetration through blood-brain barrier in CNS. It is usually bacteriostatic but it has bactericidal action against *Listeria monocytogenes,* when this acts like an intracellular pathogen.

Interestingly, Chen and colleagues in a recent study on adult patients observed that the majority of patients with hydrocephalus had delayed recognition, and did not receive targeted antibiotic treatment during the early stage of *Listeria monocytogenes* meningitis management [14].

Indeed, diagnosing Listeria meningitis poses a tough challenge and adequate therapy is frequently delayed. Firstly, it is known that clinical presentation of Listeria meningitis is similar to that of other viral and bacterial CNS infections and that Listeria resists to first-line treatment for bacterial meningitis with extended-spectrum cephalosporins. Moreover, *Listeria monocytogenes* on CSF examination can be pleomorphic (coccus, coccobacillus, bacillus, diplococcus or difteroides) and Gram stain can result negative in about half of cases. In our case, we detected positivity for bacillus bacteria that should not be automatically considered as a contaminant. PCR represents the gold standard for a quick identification in the first 24 h [15], but PCR is not widespreadly available.

Regarding pediatric cases complicated by hydrocephalus, all subjects received invasive procedures for hydrocephalus management. Of note, our patient needed a two-stage neurosurgical intervention, introducing a catheter in both ventricles and connecting them to shunt valve internalized in peritoneum. On the other side, the clinical outcome was good with a considerable neurological recovery and no particular sequelae except for an abduction deficit in the right eye (lateral rectus palsy). The same residual neurological deficit was described in patient 4, while patients 2 and 10 were reported to have development delay and tremor with adiadochokinesis, respectively. Patients 8 and 9 died, while the remaining four patients had no relevant sequelae.

Our case emphasizes the importance of suspecting early hydrocephalus as a complication of Listeria meningitis in previously healthy children with neurological deterioration few days after initial presentation, even if adequate antibiotic treatment has been started.

Poor data are present in the literature about management and outcome of Listeria meningitis-related hydrocephalus in children. It is important for a pediatrician to perform close clinical monitoring of these children and provide aggressive critical care interventions. Conservative treatment is often employed but invasive procedures are mandatory in patients with moderate or severe hydrocephalus and clinical decay.

## Data Availability

Not applicable.

## Abbreviations

WBC: White blood cells
CRP: C-reactive protein
PCT: Procalcitonin
MRI: Magnetic resonance imaging
CSF: Cerebral-spinal fluid
RT-PCR: Real-time polymerase chain reaction
CT: Computed tomography
TPM-SMX: Trimethoprim/sulfamethoxazole
